# Mesh Repair Versus No-Mesh Repair for the Management of Acute and Elective Femoral Hernias: A Systematic Review and Meta-Analysis of Perioperative Outcomes

**DOI:** 10.7759/cureus.98886

**Published:** 2025-12-10

**Authors:** Ahmed Abdalgany, Hashem Malkawi, Mohamed Abdelgawad, Goldie Khera, Muhammad Sajid

**Affiliations:** 1 General Surgery, West Suffolk NHS Foundation Trust, Bury St Edmunds, GBR; 2 Gastrointestinal Surgery, Royal Sussex County Hospital, Brighton, GBR; 3 Digestive Disease and General Surgery, Royal Sussex County Hospital, Brighton, GBR; 4 Surgery, Royal Sussex County Hospital, Brighton, GBR

**Keywords:** femoral hernia, mesh repair, no-mesh repair, recurrence, surgical site infection

## Abstract

The objective of this meta-analysis was to compare mesh repair versus no-mesh repair in the management of acute and elective femoral hernia repair. Different studies comparing mesh repair versus no-mesh repair in the management of femoral hernia were selected from medical electronic databases, and a meta-analysis was conducted in accordance with the Cochrane Collaboration guidelines using statistical software RevMan version 5.4 (The Cochrane Collaboration, London, UK). Four retrospective studies and one prospective study were included, involving 537 patients, reporting the incidence of recurrence, surgical site infection, complications, and length of hospital stay. In the random effects model analysis, the length of hospital stay was lower in the mesh group but with significant statistical heterogeneity (standardized mean difference (SMD) -0.34, 95% CI (-1.78, -1.11), Z = 0.46, P = 0.65). However, the variables of hernia recurrence, surgical site infection, and total complications were in favor of the mesh group, despite no statistically significant difference between both groups and without any statistical heterogeneity among the included studies: (risk ratio (RR) 0.50, 95% CI (0.25, 1.02), Z = 1.91, P = 0.06); (RR 0.95, 95% CI (0.35, 2.57), Z = 0.10, P = 0.92); (RR 0.99, 95% CI (0.56, 1.74), Z = 0.05, P = 0.96). This systematic review indicates that mesh repair of the femoral hernia does not offer any advantage over no-mesh repair for recurrence. Due to the paucity of randomized clinical trials and significant heterogeneity among the compared variables, a major multicenter randomized clinical trial is needed to validate these findings.

## Introduction and background

Femoral hernia occurs due to an increase in intra-abdominal pressure, which subsequently leads to widening of the femoral ring and the protrusion of a viscus or part of a viscus. This condition may cause various symptoms, including discomfort, swelling, pain, constipation, obstruction, or strangulation [1]. Diagnosis of a femoral hernia can be achieved through physical examination, ultrasonography, or computed tomography; however, it may be misdiagnosed as an inguinal hernia, which is more common. The incidence of femoral hernia is not common, representing 2-4% of all groin hernias [2]. In the United Kingdom, about 5,000 repairs of femoral hernias are performed annually, with an average cost of £3,870 [3], and the condition has been reported to be more common in female patients [4]. Despite their rarity, femoral hernias are associated with a high rate of complications, including obstruction, strangulation, bowel resection, prolonged hospital stays, chronic groin pain, and wound infection, with a morbidity rate ranging from 30% to 60% [5]. Emergency repair of the femoral hernia is linked to a mortality rate of up to 10%, according to the literature [5]. The rate of recurrence of femoral hernias is higher in male patients (16-21%) than in female patients (5-6%), based on two national databases in the United States that collected data between 2005 and 2015 [2]. Many surgical approaches have been reported for femoral hernia repair, including infrainguinal, transinguinal, preperitoneal, transperitoneal, and laparoscopic approaches. Techniques such as the mesh plug and McVay technique (the classic method for femoral hernia repair) are widely used. However, the McVay technique has been associated with recurrence rates as high as 15% [6,7].

Currently, recommendations for the optimal method of femoral hernia repair remain vague. Some guidelines recommend avoiding the use of mesh, particularly in emergency hernia repairs, due to potential contamination of the wound and risk of infection. However, the use of mesh is associated with a lower incidence of seroma and may be suitable for low-income countries where cost is a significant factor. Other studies support the use of mesh repair, citing its advantages in lowering the rate of recurrence and being less likely to be complicated by neurovascular damage. Additionally, mesh can be used in emergency cases, with the risk-to-benefit ratio favoring its use in such settings [8,9]. The objective of this review was to compare mesh repair versus no-mesh repair for the management of both acute and elective femoral hernias.

## Review

Methods

Data Sources and Literature Search Technique 

PubMed, Embase, MEDLINE, and the Cochrane Library were searched comprehensively. The term “mesh” was used to identify relevant studies, and the patient, intervention, comparison, and outcome (PICO) methodology was applied. Boolean operators (AND, OR) and femoral hernia phrases (e.g., femoral hernia repair, no-mesh hernia repair) were used to refine the search results. Additionally, references of the selected studies were examined to identify additional relevant publications. 

Trial Selection 

Inclusion criteria: Trial studies that compared mesh repair to no-mesh repair for femoral hernias and reported perioperative outcomes were deemed to meet the inclusion criteria for the systematic review. All trials, regardless of the number of patients, were considered appropriate for inclusion.

Exclusion criteria: Articles that included all groin hernias (inguinal and femoral), articles that did not compare mesh repair versus no-mesh repair for femoral hernias, and articles published in languages other than English were excluded.

Data Collection and Management 

The published data were sought and collected by the authors independently using a pre-planned standard data extraction sheet. The acquired data were reviewed by all authors to identify any disagreements, and a mutual agreement was reached regarding their accuracy. The primary variables for data collection included the list of published authors, the country in which the included studies were conducted, the year of publication, demographic information of the study population, the number of hernia recurrences, the length of hospital stay, surgical site infections, total complications, and the duration of the operation.

Evidence Synthesis Using RevMan Statistical Software 

Statistical analysis was conducted using RevMan version 5.4 (The Cochrane Collaboration, London, UK) [10]. Risk ratios (RRs) were used to present the results of continuous variables (e.g., wound complications and hernia recurrences), while standardized mean differences (SMDs) were used for dichotomous variables, such as the length of hospital stay and operation time. Under the random-effects model analysis, SMD and RR were computed and displayed with a 95% CI [11,12]. The data were presented graphically using forest plots. The I² test was used to quantify heterogeneity, with a maximum value of 30% indicating minimal heterogeneity. The chi-square test was used to assess statistical heterogeneity, with significance set at P < 0.05 [13]. The random-effects model analysis employed the inverse-variance approach to calculate SMD and the Mantel-Haenszel method to calculate RR [14,15]. The standard deviation was estimated from the range or p-value, or 0.5 was added to the cell frequency, assuming the same variance in all groups, which may not always be the case, if it was not stated in the published articles on the studies. Based on the impact weights in the results established by the experimental estimate variance, the estimate of the difference between the two approaches was combined.

Quality of Analysis 

The quality of the included studies was assessed using the National Heart, Lung, and Blood Institute (NHLBI) Quality Assessment Tool for non-randomized comparative studies [15].

Selected Endpoints for Analysis 

The primary endpoint for this study was the recurrence of a femoral hernia at the conclusion of the follow-up period. Recurrence was identified through clinical evaluation by a senior surgeon, radiological diagnostic examination, and symptomatic presentation of a recurring lump at the site of prior surgery. Secondary objectives included the length of hospital stay, operation time, and surgical site infections.

Result

**Figure 1:**
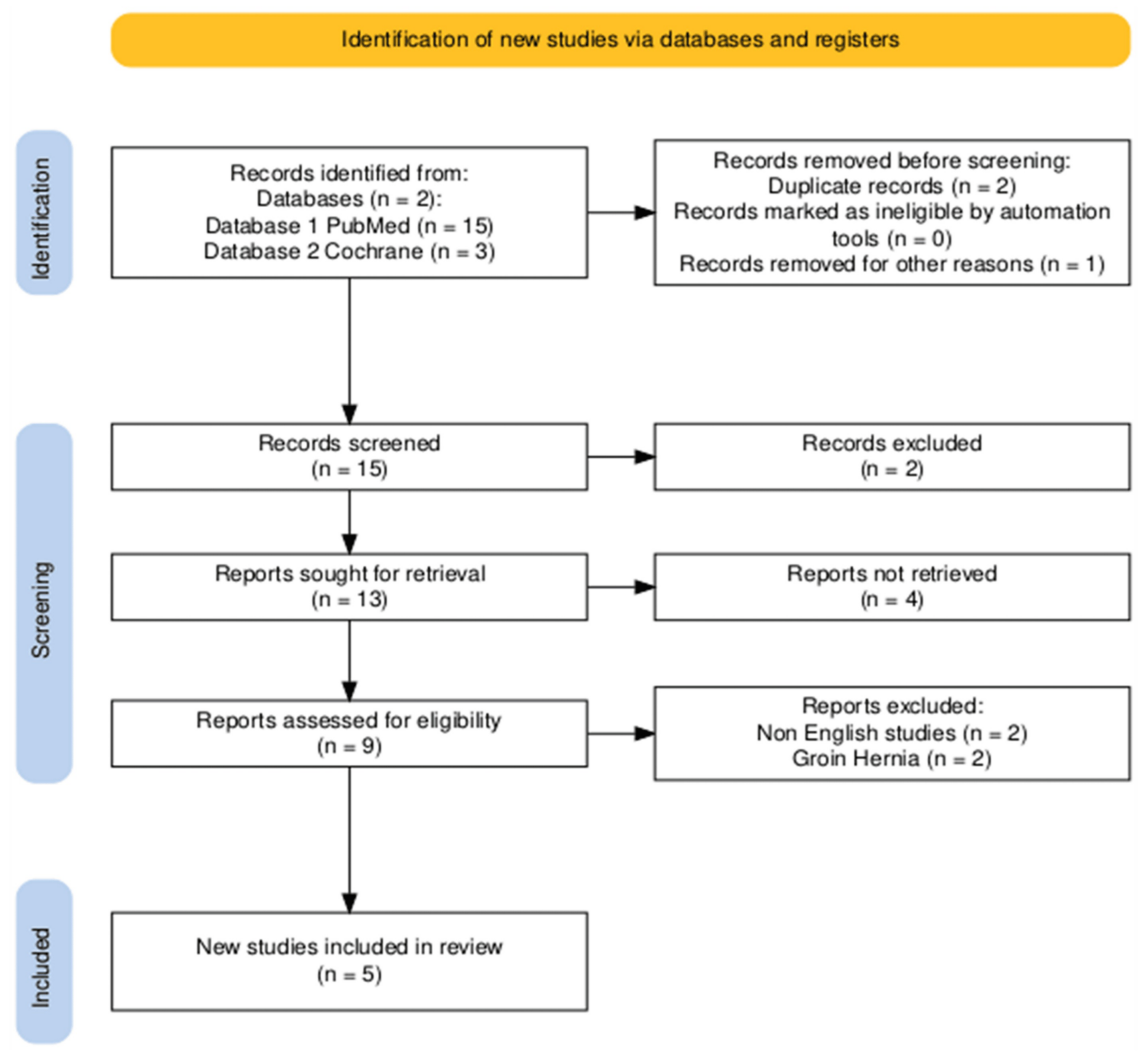
PRISMA flowchart showing literature search outcomes PRISMA: Preferred Reporting Items for Systematic Reviews and Meta-Analyses; Cochrane: Cochrane Library

The primary search of four medical databases (PubMed, Embase, MEDLINE, and the Cochrane Library) yielded 18 potentially eligible studies, all from PubMed (n = 15) and the Cochrane Library (n = 3). No eligible studies were identified in Embase or MEDLINE. After multiple stages of screening, 13 studies were excluded; the reasons for exclusion are detailed in the Preferred Reporting Items for Systematic Reviews and Meta-Analyses (PRISMA) flowchart (Figure 1) [16].

The characteristics of the included studies are presented in Table 1, and the treatment protocols for the included studies are described in Table 2.

**Table 1:** Characteristics of the included studies BMI: body mass index; M: male; F: female

Study	Country	Type of study	Follow-up duration (months)	Age (years)	Gender M:F	BMI (kg/m²)
Acar et al., 2020 [1]	Turkey	Retrospective	49.4	64.6 ± 18.3	13:35	Not reported
Liu et al., 2020 [2]	China	Retrospective	24	Mesh: 72.4 ± 10.1, No-mesh: 72.5 ± 13.0	71:33	Mesh: 18.3 ± 2.5, No-mesh: 18.9 ± 1.8
Clyde et al., 2020 [4]	UK	Retrospective	60-93	Mesh: 56.2 ± 14.1, No-mesh: 63.0 ± 16.2	38:66	Not reported
Chan et al., 2008 [6]	Canada	Prospective	60	55 (14.7-87.5)	121:104	23.8 (15.4-34.9)
Li et al., 2023 [8]	China	Retrospective	4-59	Mesh: 70.94 ± 9.28, No-mesh: 74.44 ± 10.72	12:36	Mesh: 20.39 ± 2.92, No-mesh: 20.31 ± 3.48

**Table 2:** Treatment protocol adopted in the included studies

Study	Mesh group	No-mesh group
Acar et al., 2020 [1]	Both emergency and elective femoral hernia cases. Open pre-peritoneal approach. Prosthetic polypropylene mesh plug. Placed into the femoral canal, then sutured with non-absorbable sutures in three quadrants.	Both emergency and elective femoral hernia cases. Open approach. McVay repair was performed with interrupted non-absorbable sutures between the aponeurotic margin of the transverse abdominal muscle and Cooper’s ligament.
Liu et al., 2020 [2]	Emergency femoral hernia cases. Open pre-peritoneal approach. UHS mesh (bilayer polypropylene). The mesh was fixed with absorbable sutures to the pubic tubercle and the inguinal ligament.	Emergency femoral hernia cases. Open inguinal approach. McVay repair was adopted. The conjoint tendon was sutured to the pectineal ligament.
Clyde et al., 2020 [4]	Both emergency and elective femoral hernia cases. Laparoscopic repair with pre-peritoneal mesh. Type of mesh not reported. The mesh was fixed with sutures.	Both emergency and elective femoral hernia cases. Low approach in 26 cases, higher approach in 30 cases. Performed using non-absorbable, monofilament polypropylene sutures to approximate Cooper’s ligament to the inguinal ligament.
Chan et al., 2008 [6]	Both emergency and elective femoral hernia cases. Open pre-peritoneal approach. Polypropylene mesh. The mesh was fixed with a simple interrupted 2-0 polypropylene suture.	Both emergency and elective femoral hernia cases. Open inguinal approach. Complete groin repair with a non-absorbable suture of 2-0 polypropylene.
Li et al., 2023 [8]	Emergency femoral hernia cases. Open pre-peritoneal approach. UHSL (Johnson and Johnson, USA) was used in eight patients, Shanshi D8 (Transeasy, China) was used in six patients, and biological mesh (COOK, USA) was used in two patients. Mesh fixation technique not reported.	Emergency femoral hernia cases. Open approach. Patients underwent McVay repair, which was performed with interrupted non-absorbable sutures placed between the aponeurotic margin of the transverse abdominal muscle and Cooper’s ligament.

Methodological Quality of the Included Studies 

The quality assessment variables used for assessing the strength of the included comparative studies are summarized in Table 3. One study was rated as high quality with a score of nine out of 12 [2]. The remaining included studies were considered to be of moderate quality, with scores ranging from six to eight out of 12 [1,4,6,8].

**Table 3:** Quality assessment of the included studies N/A: not applicable

Study	Were the people assessing the outcomes blinded to the participants' exposures/interventions?	Was the loss to follow-up after baseline 20% or less? Were those lost to follow-up accounted for in the analysis?	Did the statistical methods examine changes in outcome measures from before to after the intervention? Were statistical tests done that provided p-values for the pre-to-post changes?	Were outcome measures of interest taken multiple times before the intervention and multiple times after the intervention (i.e., did they use an interrupted time-series design)?	If the intervention was conducted at a group level (e.g., a whole hospital, a community, etc.), did the statistical analysis consider the use of individual-level data to determine effects at the group level?	Were the participants in the study representative of those who would be eligible for the test/service/intervention in the general or clinical population of interest?	Were all eligible participants who met the pre-specified entry criteria enrolled?	Was the sample size sufficiently large to provide confidence in the findings?	Was the test/service/intervention clearly described and delivered consistently across the study population?	Were the outcome measures pre-specified, clearly defined, valid, reliable, and assessed consistently across all study participants?	Was the study question or objective clearly stated?	Were the eligibility/selection criteria for the study population pre-specified and clearly described?	Score
Acar et al., 2020 [1]	N/A	Yes	Yes	No	No	Yes	Yes	No	No	Yes	Yes	Yes	6/12
Liu et al., 2020 [2]	N/A	Yes	Yes	No	No	Yes	Yes	Yes	Yes	Yes	Yes	Yes	9/12
Clyde et al., 2020 [4]	N/A	No	Yes	No	No	Yes	No	Yes	Yes	Yes	Yes	No	6/12
Chan et al., 2008 [6]	N/A	Yes	Yes	No	No	Yes	No	Yes	Yes	Yes	Yes	No	7/12
Li et al., 2023 [8]	N/A	Yes	Yes	No	No	Yes	Yes	No	Yes	Yes	Yes	Yes	8/12

Primary Outcome Analysis 

**Figure 2:**
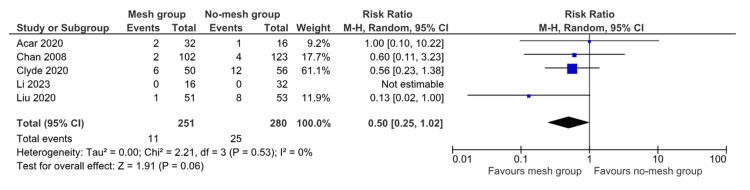
Forest plot showing the risk of recurrence in the mesh group versus the no-mesh group. The outcome is presented as a risk ratio with a 95% confidence interval. [1,2,4,6,8]

In the random effects model analysis, femoral hernia recurrence was found to be statistically similar between the mesh group and the no-mesh group (RR 0.50, 95% CI (0.25, 1.02), Z = 1.91, P = 0.06) (Figure 2). There was no heterogeneity among the included studies (Tau² = 0.00; Chi² = 2.21, df = 3; P = 0.53; I² = 0%).

Secondary Outcome Analysis

**Figure 3:**
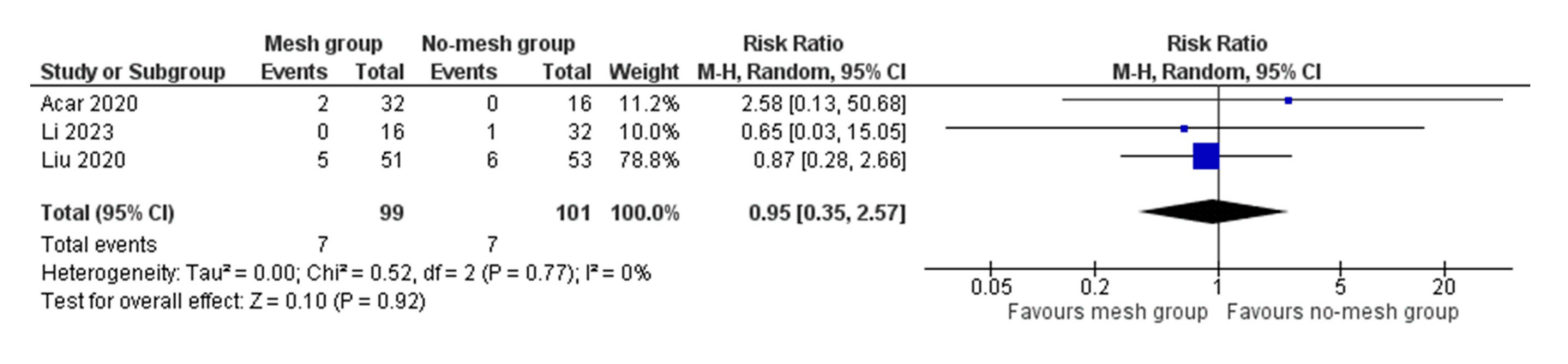
Forest plot showing the risk of surgical site infection in the mesh group versus the no-mesh group. The outcome is presented as a risk ratio with a 95% confidence interval. [1,2,8]

In the random effects model analysis, the risk of surgical site infection showed no statistically significant difference between the mesh group and the no-mesh group (RR 0.95, 95% CI (0.35, 2.57), Z = 0.10, P = 0.92) (Figure 3). There was no heterogeneity among the included studies (Tau² = 0.00; Chi² = 0.52, df = 2; P = 0.77; I² = 0%).

**Figure 4:**
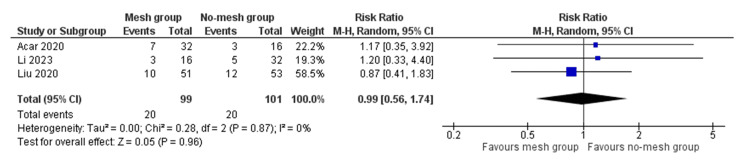
Forest plot showing the risk of complications in the mesh group versus the no-mesh group. The outcome is presented as a risk ratio with a 95% confidence interval. [1,2,8]

**Figure 5:**
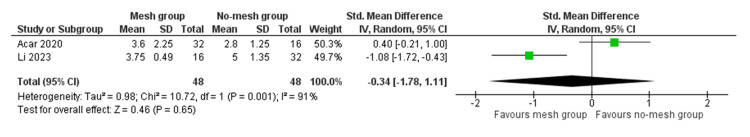
Forest plot showing the length of hospital stay in the mesh group versus the no-mesh group. The outcome is presented as a risk ratio with a 95% confidence interval. [1,8]

The risk for other complications (chronic groin pain, seroma, delayed wound healing) was lower in the mesh group compared to the no-mesh group; however, there was no statistically significant difference, and there was no heterogeneity (RR 0.99, 95% CI (0.56, 1.74), Z = 0.05, P = 0.96) (Tau² = 0.00; Chi² = 0.28, df = 2; P = 0.87; I² = 0%) (Figure 4). Moreover, the length of hospital stay was shorter in the mesh group, but this result showed significant statistical heterogeneity (SMD -0.34, 95% CI (-1.78, -1.11), Z = 0.46, P = 0.65) (Tau² = 0.98; Chi² = 10.72, df = 1; P = 0.001; I² = 91%) (Figure 5).

Discussion

Summary of Findings 

This meta-analysis of five different studies [1,2,4,6,8], including both retrospective and prospective studies, demonstrated no statistically significant difference in the recurrence rate of patients undergoing femoral hernia repair with mesh versus suture repair. Although surgical site infection was a concern due to the presence of the prosthetic mesh material, this was not evident in the meta-analysis from the data reviewed, and infection rates were not significantly higher in the mesh group compared to the no-mesh group. Overall complication rates were low in both groups, with no statistically significant difference. Similarly, the available data on the length of hospital stay and operative time revealed no differences between the groups.

Comparison With Existing Literature 

The observed benefits of mesh reinforcement in femoral hernias align poorly with the established evidence from prior studies in inguinal and ventral hernia repairs. Previous studies, such as those by the European Union (EU) Hernia Trialists Collaboration, have demonstrated a significantly reduced recurrence rate of femoral hernias with mesh repair [9]. From our study, the RR favors the mesh group; however, there is no statistically significant difference, with a p-value of 0.53. Accordingly, the choice of repair type remains at the surgeon’s discretion.

Historically, concerns about increased infection risk have discouraged the use of mesh in femoral hernia surgery, specifically in elderly patients or emergencies involving bowel ischemia. However, the data reviewed did not support an elevated statistically significant surgical site infection with mesh repair.

Furthermore, the low infection rates observed in patients treated with mesh are likely due to adherence to aseptic protocols and patient selection. These findings could indicate that the risk of infection may depend more on patient comorbidities and intraoperative conditions rather than the use of the mesh itself. Comparisons with other prior systematic reviews are limited by the rarity of studies focusing exclusively on femoral hernia repair.

Clinical and Surgical Implications 

The results of this review have significant implications for clinical practice. Femoral hernias, particularly in women, are associated with higher risks of incarceration and strangulation. Given the lack of significant difference in recurrence or complication rates, both mesh and suture repair may be considered, depending on the surgeon’s preference.

In emergency or contaminated settings, mesh use requires a more individualized and tailored approach. Factors such as the extent of contamination, bowel viability, and patient-specific risks must inform surgical decision-making.

Future research should prioritize well-designed, prospective randomized clinical trials comparing mesh and no-mesh repair in femoral hernias, in addition to adopting standardized outcomes, including clinical, functional, and patient-reported endpoints. Furthermore, analyses should be based on patient risk factors (e.g., age, sex, comorbidities) and surgical context (elective vs. emergency), including cost-effectiveness data to inform surgical decision-making in diverse healthcare settings. Evaluation of biologic versus synthetic mesh materials and providing long-term follow-up (≥ 5 years) to assess recurrence, chronic pain, and quality of life are crucial. Establishing a collaborative registry or multicenter database for femoral hernia repairs would significantly enhance the quality and scope of available evidence.

Strengths 

This review is among the few that focus solely on femoral hernia repairs, providing valuable insight into a frequently overlooked area of hernia surgery. It includes both retrospective and prospective studies from multiple geographic regions, enhancing the external validity of its conclusions. The analysis covers a wide range of outcomes - recurrence, surgical site infections, complications, length of hospital stay, and operating time - offering a thorough assessment of the comparative efficacy and safety of mesh repair and suture repair. Recent studies were incorporated to ensure contemporary relevance. Additionally, the use of forest plots for major outcomes enhances interpretability and transparency, offering a clear visualization of trends and effect sizes across studies.

Limitations 

Several limitations must be considered. The predominance of retrospective designs introduces potential biases, including selection and reporting bias. Only one prospective study, four retrospective studies, and no randomized controlled trials were identified. There was marked heterogeneity in outcome definitions and reporting practices, particularly regarding complications, length of hospital stay, and operating time. Key patient characteristics, such as body mass index (BMI), American Society of Anesthesiologists (ASA) physical status classification, smoking status, and diabetes, were inconsistently reported, preventing a more stratified analysis. Furthermore, sample sizes in several studies were small, limiting the power to detect differences in less frequent outcomes, such as surgical site infections. Follow-up periods also varied considerably, potentially influencing the detection of recurrence over time. Publication bias is another concern, as studies with null or negative results may be underrepresented. Finally, language and database restrictions may have excluded relevant studies not indexed in English-language sources.

## Conclusions

This systematic review indicates that the RR for recurrence favored the use of mesh, although it was not statistically significant. Due to the paucity of randomized clinical trials and significant heterogeneity among the compared variables, a major multicenter randomized clinical trial is needed to validate these findings.
